# Histotripsy Dose Impacts Treated Tumor Immune Infiltration and Survival Outcomes in a Murine B16F10 Melanoma Model

**DOI:** 10.3390/cancers17233773

**Published:** 2025-11-26

**Authors:** Reliza McGinnis, Brian Song, Hanna Kim, Anna Lorenzon, Jiaqi Shi, Lili Zhao, Clifford S. Cho, Anutosh Ganguly, Zhen Xu

**Affiliations:** 1 Department of Biomedical Engineering, University of Michigan, Ann Arbor, MI 48109, USA; remcginn@umich.edu (R.M.); hahkim@umich.edu (H.K.); alorenzo@umich.edu (A.L.); 2 Department of Surgery, University of Michigan, Ann Arbor, MI 48109, USA; brisong@umich.edu (B.S.); clifford.cho@vai.org (C.S.C.); ganutosh@umich.edu (A.G.); 3 Department of Pathology, University of Michigan, Ann Arbor, MI 48109, USA; jiaqis@med.umich.edu (J.S.); zhaolili@umich.edu (L.Z.); 4 Department of Cell Biology, Van Andel Institute, Grand Rapids, MI 49503, USA; 5 Department of Radiology, University of Michigan, Ann Arbor, MI 48109, USA; 6 Department of Neurosurgery, University of Michigan, Ann Arbor, MI 48109, USA

**Keywords:** histotripsy, cancer immunology, FUS, dose study, B16F10 melanoma

## Abstract

**Background/Objectives:** Preclinical animal studies and clinical case reports have shown evidence of histotripsy being capable of inducing anti-tumor immune responses strong enough to inhibit tumor growth of off-target tumors. Previous studies exploring histotripsy immune stimulation have used a single therapy dose. This study aims to explore how histotripsy dose affects local tumor immune stimulation in a murine melanoma model. **Methods:** C57BL/6J mice bearing subcutaneous B16F10 tumors were treated with histotripsy using an ultrasound-guided 8-element 1 MHz transducer operating at a 100 Hz pulse repetition frequency (PRF) and >30 MPa peak-negative pressure. The histotripsy dose was defined by the number of pulses (8, 20, 40, or 100) per focal location (ppl). Tissue damage and residual tissue structure were measured histologically and scored by a trained pathologist. The longitudinal effect of histotripsy dosing was assessed using tumor growth and survival. Acute immune stimulation was measured at days 2 and 7 post-treatment via immunofluorescence staining of the treated tumor. **Results:** Histotripsy doses at 20, 40, and 100 ppl achieved significant tumor necrosis within the target region (>75%), with residual structure decreasing as the dose increased. Overall, the greatest tumor control was observed in mice that received the 40 ppl dose compared to untreated mice. This correlates with the 40 ppl dose also having the largest increase in CD45+ immune cells and CD8^+^ T cells 7 days post-treatment compared to untreated mice. **Conclusions:** The effect of histotripsy dosing on immune infiltration and tumor growth highlights the significant impact of histotripsy dose on clinical effects.

## Simple Summary

Histotripsy is a non-invasive ultrasound treatment that mechanically breaks down tumor tissue without using heat or radiation. Recently approved by the FDA for liver tumor treatment, this technique shows promising potential beyond local tumor control by stimulating an anti-tumor immune response. Histotripsy has repeatedly been demonstrated for safe and effective ablation; however, there is a remaining gap in the knowledge regarding how therapy dose impacts immune stimulation. This study aims to determine the impact of histotripsy dose on animal survival and tumor progression while also exploring how therapy dose affects subsequent immune stimulation. Understanding the impact of histotripsy therapy dose on immune stimulation is critical for aiding researchers and clinicians towards developing more effective and precise histotripsy cancer treatment procedures that could harness the body’s own immune system to fight cancer.

## 1. Introduction

Histotripsy’s recent FDA approval in October 2023 for non-invasive treatment of liver tumors marks an exciting milestone in the field of therapeutic ultrasound [1,2]. Histotripsy uses high-pressure, microsecond-length, repeating pulses of focused ultrasound to non-invasively generate inertial cavitation in targeted tissue. The cavitation mechanically disrupts cells and fractionates the targeted tissue without using ionizing energy or generating significant heat at the target and surrounding tissue [3,4]. In addition to local tumor control, published rodent pre-clinical studies have shown that histotripsy mono-therapy ablation of primary tumors can lead to arrested growth and even remission of systemic untreated tumors [5]. This systemic anti-tumor response is typically referred to as the ‘abscopal’ response and is a major focus of research for its implications for human metastatic cancers [6,7]. It has been shown that the mechanical tissue damage instigated by histotripsy causes the release of damage-associated molecular patterns (DAMPs) into the extracellular space, as well as evidence of transiently increased necroptotic cell death, peaking two days post-treatment, in surviving tumor cells at the boundary of the treated lesion. This early inflammatory event is correlated with early neutrophil, macrophage, and NK cell infiltration into the surrounding remaining tumor, which eventually develops into a CD8^+^ cytotoxic T lymphocyte (CTL)-dominated response when treating subcutaneous murine B16-F10 melanoma tumors and Hepa1-6 liver tumors. These infiltrating CTLs appear to be tumor-antigen specific and capable of directly killing tumor cells via known pathways, such as ferroptosis (known to be an important mechanism of CTL anti-tumor function) [8,9,10,11]. This abscopal response has even been reported in individual late-stage human patients who received histotripsy therapy to liver tumors, highlighting histotripsy’s potential for improving patient outcomes [12].

A glaring hole in our understanding of histotripsy immune stimulation, however, is the effect of histotripsy dose on the landscape and magnitude of the local immune response. Previous studies have explored the general safety and dose necessary to cause complete cellular fractionation the target tissue (verified through H&E staining), but it is still unclear how varying this dosage affects immune cell recruitment to the locally treated tumor [13,14,15].

In histotripsy treatment, each ultrasound pulse breaks down a portion of the tumor cells, and an accumulation of the ultrasound pulses eventually liquefies the target tumor to acellular debris [16]. Therefore, the histotripsy dose is defined as the number of ultrasound pulses delivered at each targeted location (ppl). At low doses of histotripsy, targeted tumors cells are only partially disrupted. With increasing doses, more cells are ruptured and tissues fractionated, releasing contents like nuclei and peptides (including tumor antigens) extracellularly [17]. At higher histotripsy doses, both the tumor cell membrane and all cell nuclei are completely destroyed [18]. We hypothesize that an excessively high therapy dose would negatively impact the exposure of tumor antigen to the immune system after histotripsy. Therefore, we also hypothesize that an intermediate histotripsy dose that induces complete tumor cell membrane disruption with sufficient extracellular release of intact cellular content will result in higher antigen-guided tumor immune cell infiltration and immune response compared with lower doses that produce partial tissue damage, or very high doses beyond what is necessary for complete tissue fractionation. In this study, we explore the impact of histotripsy dose on local tumor immune cell infiltration, tumor growth kinetics, and animal survival in a subcutaneous murine B16F10 melanoma model (simply referred to as tumor for the remainder of this paper).

## 2. Materials and Methods

### 2.1. Mice, Cell Line, Inoculation, and Measurement

Female C57BL/6NTac mice (Taconic farms) were housed in a pathogen-free, well-ventilated environment with regular monitoring. B16F10 cells (#CRL-6475, ATCC, Manassas, VA, USA) were cultured with sterile RPMI media supplemented with 10% fetal bovine serum. Cells were passaged when culture reached ~80% confluency. Before inoculation, the cells were treated with 0.25% trypsin (Gibco, Waltham, MA, USA) and suspended at a final concentration of 50,000 cells per 50 μL in a 1:1 volume of sterile 1x PBS and cold phenol-red free, growth factor-reduced Matrigel (Corning, Corning, NY, USA). Subcutaneous flank inoculations (bilateral or unilateral for immune profiling and survival experiments, respectively) were performed using 1 mL 26-gauge insulin syringes that remained on ice until ready for tumor cell aspiration and injection. The animals were algorithmically randomized into treatment groups on the day of treatment. Tumors were measured daily in two dimensions using digital calipers, and their volume was estimated with the smaller dimension = width and larger = length (Equation (1)) [19,20]. Mice were treated once tumors reached 5–8 mm in the largest dimension, with tumors outside of this range excluded from the study.

(1) $$Tumor\;volume=\left(length\times {width}^{2}\right)/2$$

### 2.2. Ultrasound-Guided Histotripsy Therapy

All animal treatments were performed using similar methods as our previously reported studies [10,11]. To briefly summarize, therapy was performed using a custom-built, eight-element, 1 MHz focused ultrasound therapy transducer with a 1.5-cycle pulse duration. Using a motorized positioning system, the tumor was scanned to defined treatment boundaries in three orthogonal dimensions using a L40-8/12 20 MHz imaging probe (Ultrasonix, Vancouver, BC, Canada) coaxially aligned with the therapy transducer. Boundary coordinates were used to generate a three-dimensional ellipsoid consisting of uniformly spaced treatment locations (spacing remained fixed at 0.5 mm in all dimensions). The targeted volume varied between the samples to compensate for differences in tumor volume/shape across animals. During treatment planning, the transducer focus was mechanically steered to the center cross-section of the tumor, and from there, the width and height boundaries were set by moving the transducer focus to the edge of the tumor boundary. Importantly, prescribed dimensions intentionally avoided sensitive tissue, like muscle and skin, in accordance with animal protocol guidelines for minimizing mouse harm. Due to this restriction, complete tumor treatment was not possible, and a portion of tumor (excluding the tumor at the skin boundary) was treated. The prescribed target tumor dimensions were compared to the actual tumor dimensions measured on the ultrasound imager. The length boundaries were set in the same way as the previous dimensions, but because of the limitations of using a fixed-position linear imaging transducer, we could not make similar measurements of the actual length by ultrasound. The targeting percent was estimated using the tumor dimensions that could be measured with ultrasound (Equation (2)).

(2) $$Percent\;targeted=\frac{\left({width}_{prescribed}*{{height}_{prescribed}}^{2}\right)/2}{\left({width}_{measured}*{{height}_{measured}}^{2}\right)/2}\times 100$$

The average percent targeted was 58% with a standard deviation of 18.5%. Each focal location received the same number of histotripsy pulses at a 100 Hz pulse repetition frequency (PRF) with an estimated >30 MPa peak negative pressure in the free field at the focus. The therapy transducer and imaging probe were mechanically moved to each target, allowing real-time visualization of the histotripsy bubble cloud during treatments. The histotripsy dose was varied by changing the number of pulses per location (ppl) for each treatment group, with 8, 20, 40, and 100 ppl tested in this study.

### 2.3. Tumor Processing for Microscopy

At an experimental endpoint, mice were euthanized via carbon dioxide asphyxiation and secondary cervical dislocation. Tumors were resected and fixed in 10% neutral-buffered formalin for up to 48 h, stored in 70% ethanol at room temperature, and paraffin-embedded prior to tissue slicing. Embedded tumors were sectioned 3–4 micron thick, and alternating slices were left unstained for immunofluorescent staining (IF) or stained with hematoxylin/eosin (H&E) for histological damage analysis.

### 2.4. Histological Damage Scoring

Stained slides were digitally scanned at 20x magnification on a Leica Aperio AT2 and annotated to have the ablated region of the section defined. These annotated images were scored by a trained pathologist using a two metric scoring system: % necrosis in the lesion and residual structure within the lesion (Table 1). Necrosis was determined by cell death or cell disruption.

### 2.5. Immunohistochemistry Staining and Imaging

Deparaffinization/rehydration was carried out by immersing the slides twice in xylene and then a graded series of ethanol solutions: twice in 100% ethanol, then in 70%, 50%, and 30% ethanol, and finally in 100% water. For nuclear stains, a fix/perm solution was prepared by mixing fix/perm concentrate and fix/perm diluent in a 3:1 ratio. Samples were enclosed with a hydrophobic marker, and fix/perm solution was added per slide. The slides were incubated in a humid chamber at 37 °C for 20 min. After incubation, the fix/perm solution was removed, and permeabilization solution was added per slide for 20 min at room temperature (RT). The slides were then washed in PBS. Antigen retrieval was performed by immersing the slides in heated ddH_2_O-diluted citrate buffer (70–90 °C). The slides were then moved to heated ddH_2_O-diluted Tris buffer (70–90 °C). After the slides cooled in PBS, blocking was performed by adding blocking buffer (ThermoFisher, Waltham, MA, USA, 37520) to each slide and incubating at RT. The slides were then washed in PBS. For immunostaining, primary antibodies were diluted to 1:100 in universal antibody dilution buffer. The primary antibody cocktail was added to each slide, followed by incubation at 37 °C or overnight at 4 °C. After incubation, the slides were washed in PBS. Secondary antibodies were then prepared by diluting them to 1:200 in universal antibody dilution buffer. The secondary antibody cocktail was added to each slide and incubated at 37 °C, followed by a wash in PBS. For a second set of primary and secondary antibodies, 3.5% PFA was briefly added and removed, followed by a wash in PBS. The second primary and secondary antibody cocktails were prepared similarly, with primary antibodies diluted to 1:100 and secondary antibodies to 1:200. The second primary antibody was added to each slide and incubated at 37 °C, followed by a wash in PBS. The second secondary antibody was then added, incubated at 37 °C, and washed in PBS. The quenching buffer (Vector, Newark, CA, USA, SP-8400-15) was added to each slide, followed by a wash in PBS. The slides received mounting media, a cover glass, and had air bubbles removed. The slides were dried, and regions of interest (ROIs) were imaged on a Keyence BZ-800 microscope (Keyence, Osaka, Japan) or a Nikon Ti2 (Nikon, Tokyo, Japan) at 20×.

### 2.6. Survival Studies

For survival studies, mice were inoculated with unilateral, subcutaneous flank tumors and monitored/measured daily post-histotripsy treatment (n = 12–13 per group, 63 total). Mice were euthanized if any of the following criteria was met: 1 month post-inoculation, the largest dimension of a single tumor exceeded 2 cm; continued bleeding around the tumor for more than 48 h after first observation; or heavily impacted movement due to tumor burden.

### 2.7. Statistical Analysis

All statistical analysis was performed in RStudio (R version 4.3.3). Comparisons from immunofluorescent staining were made using one-way ANOVAs with repeated measures and Dunnett’s test for multiple comparisons. Survival was compared using log-rank comparison between groups with the “survminer” R library [21]. Longitudinal tumor growth between groups was compared by fitting generalized additive mixed models (GAMMs) (Equation (3)) to the log-transformed tumor volume measurements using the R package (version 0.9.9.45) MAEVE [22,23].

(3) $$y_{ijl}=a_{ij}+b_{ij}t_{ijl}+g\left(t_{ijl};\beta_{i}\right)+\epsilon_{ijl}$$

where $\left\{\left(a_{ij},b_{ij}\right),\;j=1,\ldots ,n_{i}\right\}$ are the random effects for every animal (j). $g\left(t_{ijl};\beta_{i}\right)$ is the fixed component shared across the group (i), modeled by a smooth function of time estimated by a spline with parameter vector $\beta_{i}$. The area under the curve value for the spline fits was calculated using the endpoint gain integrated in time (eGaIT) (Equation (4)) [22].

(4) $$\gamma_{i}\equiv \frac{1}{\frac{1}{2}{\left(b-a\right)}^{2}}\int_{a}^{b}\left[g_{i}\left(t\right)-g_{i}\left(a\right)\right]dt$$

All pairwise comparison tests used observations from the control (0 ppl) group for reference, with *p*-values adjusted using the ‘fdr’ method.

## 3. Results

### 3.1. Tested Histotripsy Doses Induced Considerable Tumor Disruption

**Figure 1:**
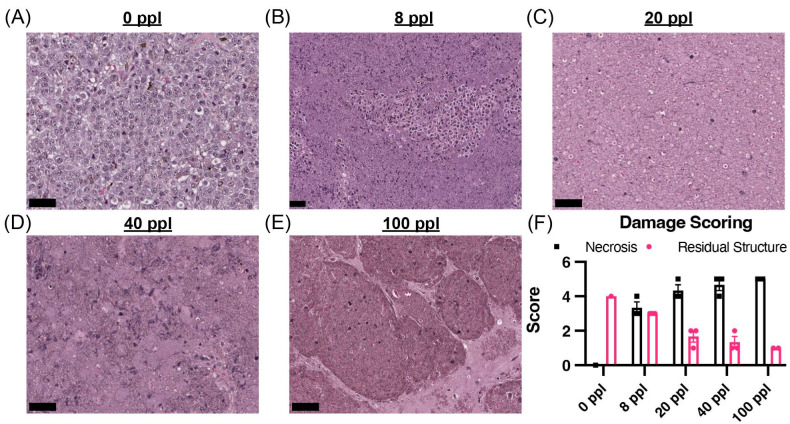
Tissue damage increased with histotripsy therapy dose in B16F10. (**A**–**E**) Representative image of the center of the therapy lesion immediately after treatment (scale bar = 50 µm). (**F**) Mean necrosis and residual structure score within the targeted therapy lesion. N = 2–3 treated tumors per group.

The extent of acute damage to targeted B16F10 tumors was evaluated based on H&E histology (Figure 1A–E). There was a total of 132 sections across 13 tumors used for acute histology. The sections that encompassed the center of the lesion and the treatment zone were then digitally scanned and scored. The purpose of the histology is to provide histological characterization of the damage effect generated by the histotripsy dose. This effect is relatively consistent across treatments, as represented by the low variance within each therapy dose.

All tested histotripsy doses induced considerable tumor disruption, with necrosis scores ranging from 3–5 (51–>95% necrosis) within the histotripsy lesions across the treated samples. The average necrosis score increased with dose, with the 8 ppl group (n = 3 mice, 9 slides total) having an average necrosis score of ~3 (51–75% necrosis), the 20 (n = 3 mice, 6 slides total) and 40 ppl (n = 3 mice, 6 slides total) groups having an average score of ~4 (76–95% necrosis), and the 100 ppl group (n = 2 mice, 5 slides total) having an average score of 5 (>95% necrosis). The residual structure had an inverse relationship, which decreased with histotripsy dose (Figure 1F).

### 3.2. Histotripsy Monotherapy Provides Dose-Dependent Survival Benefits

**Figure 2:**
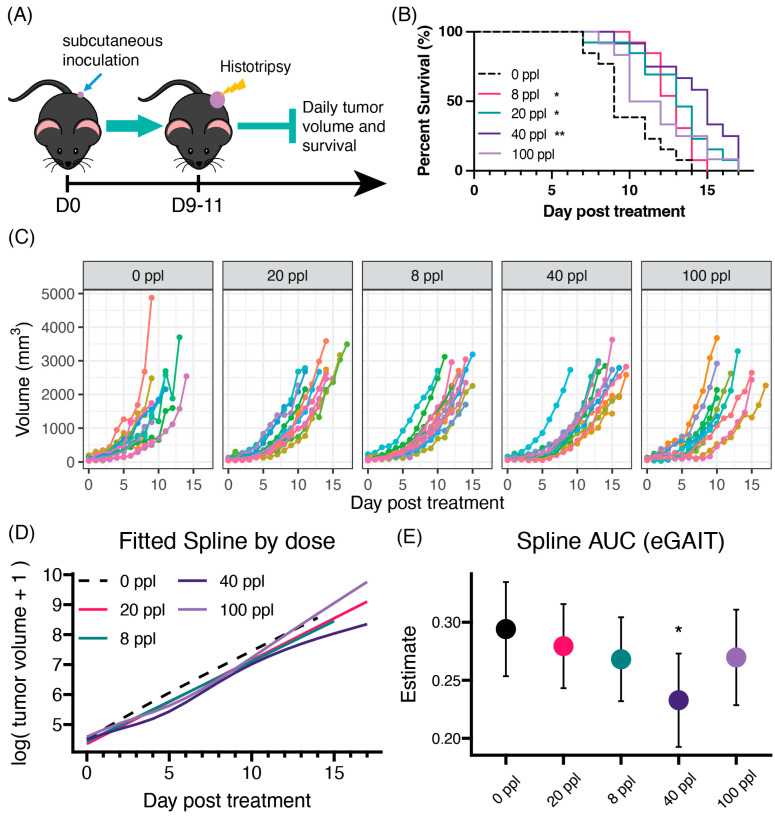
Therapy dose impacts on animal survival. (**A**) Diagram of survival study design with daily caliper tumor measurements. (**B**) Kaplan–Meier test results of overall animal survival by ppl dose. Significance measured with pairwise Mantel–Cox log rank test with shown asterisks denoting comparison to 0 ppl (control). (**C**) Individual log-transformed tumor growth post-treatment with the predicted generalized additive model spline in black. Colors represent different individuals within the dose group. (**D**) Overlay of predicted splines by dose. (**E**) Average summary eGAIT area under the curve (AUC) value for fitted splines. Error bars represent 95% CI. Significance measured by Dunnett’s pairwise comparison, with control (0 ppl) as reference. N = 12–13 per group. * *p* < 0.05, ** *p* < 0.01.

To study the impact of histotripsy dose on tumor growth and animal survival, mice bearing unilateral subcutaneous flank tumors were treated with histotripsy 8–10 days after tumor inoculation, and survival time was recorded for each mouse (Figure 2A). Mice in the 8 (n = 13), 20 (n = 13), and 40 (n = 12) ppl groups saw some overall survival benefit compared to untreated controls (n = 13), with the largest improvements in the 20 and 40 ppl groups (3 days), while mice in the 100 ppl (n = 12) group only saw marginal survival benefit (Figure 2B). Survival benefit appears largely attributed to a delay in tumor growth that lasts ~1-week post-treatment in all treatment groups (Figure 2C). However, the 40 ppl group showed the greatest longitudinal tumor control over the course of the study compared to untreated mice (Figure 2C,D). The area under the curve, defined by endpoint gain integrated over time (eGaIT), summarizes this as a single value that decreases with therapy dose. The 40 ppl group showed the largest drop in predicted eGaIT (0.192–0.273) compared to the untreated group (0.253–0.334); ranges represent the 95% CI. Mice in the 100 ppl group showed no difference in tumor growth compared to the untreated group, with a predicted eGaIT range of (0.23–0.297) (Figure 2E).

### 3.3. Dose-Dependent Change in Intra-Tumor Immune Cell Profile After Histotripsy

Immunofluorescence/IHC microscopy was used to evaluate the impact of histotripsy dose on the presence of immune cell populations outside of the histotripsy lesion in the locally treated tumor. Mice bearing bilateral tumors were treated with histotripsy at 8 ppl, 20 ppl, 40 ppl, 100 ppl, or untreated (0 ppl) and had both tumors resected either day 2 or 7 post-treatment.

### 3.4. General Immune Cell Infiltration Peaks at Intermediate Dose

**Figure 3:**
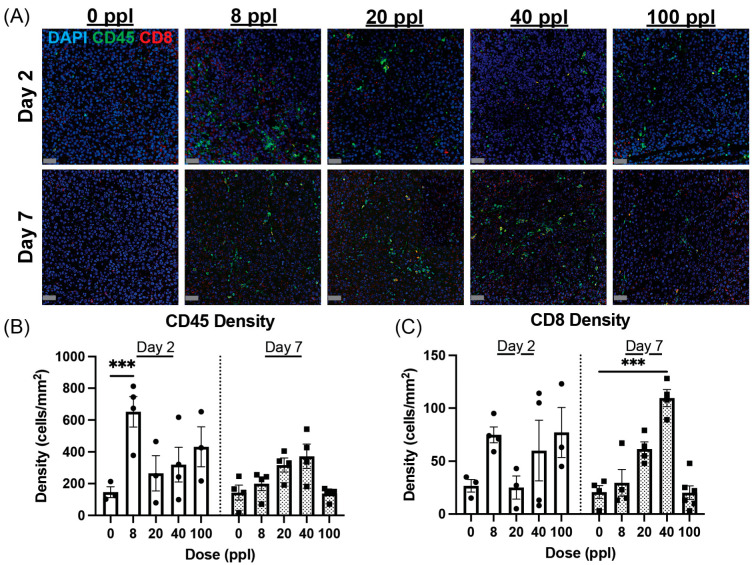
Dose-dependent trend of overall immune and CD8^+^ TILs after histotripsy. (**A**) Representative images of CD45+ immune cells (green) and CD8^+^ T cells (red) at days 2 and 7 post-histotripsy therapy, taken at 20× objective magnification. (**B**) CD45+ immune cell and (**C**) CD8^+^ TIL density were quantified in selected regions of interest in the untreated regions of tissue sections. N = 3–5 mice per group. Scale bar = 50 µm. *** *p* < 0.001.

Overall CD45+ immune cell density was slightly elevated in all treatment groups 2 days after histotripsy. The highest cell density was observed in the 8 ppl group, which had a 4× increase compared to untreated mice (Figure 3A). At day 7, CD45+ immune cell density decreased in the 8 and 100 ppl groups, while the 20 and 40 ppl groups showed a slightly elevated average compared to untreated mice (Figure 3B). Overall, immune cell density increased with therapy dose, with the 20 and 40 ppl groups having the largest increase in average cell density compared to untreated mice. Interestingly, this trend did not continue at 100 ppl, which resulted in no difference in general immune cell density compared to untreated mice.

### 3.5. CD8^+^ T-Cell Infiltration Also Peaks at Intermediate Dose

CD8^+^ T cells were measured in the locally treated tumor to gain insight into potential changes in the anti-tumor adaptive immune response after histotripsy (Figure 3C). The 40 ppl dose resulted in the highest CD8^+^ T-cell density, significantly higher than the untreated controls, at both days 2 and 7. At day 2 the 8, 40, and 100 ppl groups saw a ~2.4–3× fold increase in the average density of CD8^+^ TILs (CTL) compared to untreated mice, which had an average density of ~25 cells/mm^2^. CTL density did not change in the 20 ppl dose group at day 2. There was no change in the average CTL density in untreated tumors at day 7 compared to day 2. The 8 ppl and 100 ppl groups saw a decrease in the average CTL density at day 7 compared to day 2 (8 ppl: 75 → 32 cells/mm^2^ and 100 ppl: 77 → 22 cells/mm^2^), while the 20 and 40 ppl treatment groups saw a ~2.5× fold increase (25 → 62 cells/mm^2^) and 1.4× increase, respectively (Figure 3C). The 40 ppl dose group was the only tested dose to show a statistically significant increase (~5.2×) in CTL density at day 7 compared to untreated mice. Overall, CTL density at day 7 increased with histotripsy dose, except at the highest dose (100 ppl).

### 3.6. Dose-Dependent Decrease in F480+ Macrophage Prevalence Is Temporary

**Figure 4:**
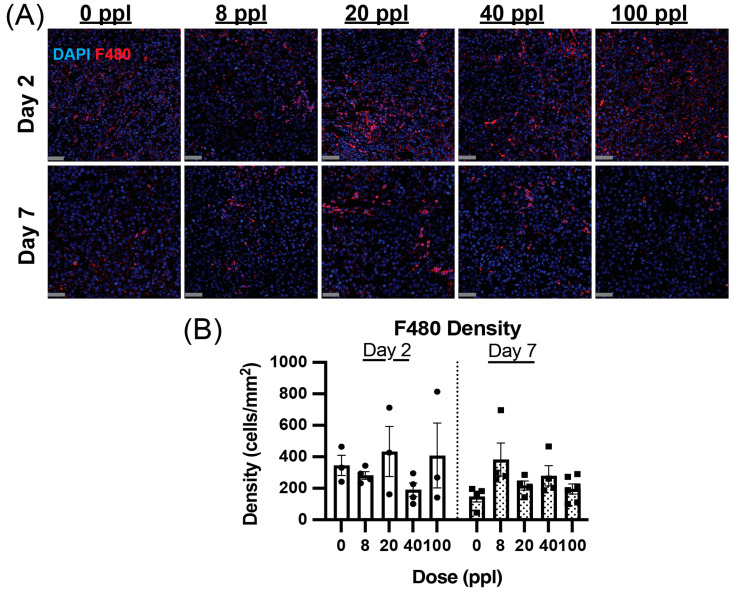
Dose-dependent change in F480+ tumor-associated macrophage prevalence. (**A**) Representative images of F480 (red) positive cells in analyzed regions of interest within stained tumor sections at days 2 and 7 post-treatment. (**B**) Sample-averaged F480+ macrophage density (cells per square mm) in analyzed ROIs. N = 3–5 mice per group. Scale bar = 50 µm.

F480+ tumor-associated macrophages (TAM) were also quantified in the residual tumor following histotripsy at days 2 and 7 post-treatment (Figure 4). Only the 40 ppl dose group saw a change in average cell density compared to the untreated group on day 2, with a ~54% decrease compared to untreated mice. On day 7, the average TAM density was the highest in the 8 ppl group compared to all other groups (Figure 4B).

## 4. Discussion

The goal of this study was to evaluate the impact of histotripsy dose (as defined by ppl) on local tumor immune infiltration, tumor growth, and animal survival in a subcutaneous B16F10 murine tumor model with partial tumor targeting (at least 50% of the tumor treated). The histotripsy doses tested in this study all induced at least 50% tumor fractionation, as defined by the ‘necrosis score’ in the targeted region. However, the lowest tested dose (8 ppl) left behind pockets of residual tissue in the targeted region. Overall, residual structure in the treated region decreased with increasing histotripsy dose. The intermediate dose (40 ppl) resulted in the best survival and tumor growth inhibition, with a decrease in F480+ TAMs and the highest CD8^+^ T-cell infiltration at days 2 and 7 post-treatment, respectively. This agreement reinforces the link between early immune activation and survival benefit following histotripsy therapy. Interestingly, the highest treated dose (100 ppl) consistently performed worse than the second highest dose (40 ppl). These results support our hypothesis that an intermediate dose that disrupts most to all tumor cells while possibly retaining substantial subcellular content, such as tumor antigen, can stimulate the immune system better than higher doses.

Tumor-associated macrophages (TAMs) are a dynamic cell population that can play immunosuppressive [24,25] or anti-tumor [26] roles in the TME; however, they are generally linked with poor prognosis when elevated in melanoma [27]. A reduction in immunosuppressive macrophages could help explain the general improved survival outcomes in the 40 ppl group, as it could signify a change in the TME to a more inflammatory state that is necessary for anti-tumor immune responses [27,28]. This highlights the importance of elucidating macrophage phenotype, which is unfortunately beyond the scope of this paper. Future studies will use as spatial transcriptomics, multiplex IHC, and advanced flow cytometry to provide more insight into the different immune cell subpopulations present after different therapy doses.

A potential reason for the non-monotonic nature of CD8^+^ density at the 20 ppl dose is due to CD8^+^ non-T-cell populations in the tumor. Dendritic cells, specifically CD8^+^CD103^+^ migratory DCs [29], could explain the early increase in CD8^+^ signaling in the 8, 40, and 100 ppl groups, while day 7 trends reflect true T cells. Given the typical kinetics of T-cell activation and infiltration, we used the early (day 2) time point to primarily analyze changes in innate immune cell populations and used the later (day 7) time point to primarily analyze changes in adaptive immune cell populations. Although CD8^+^ T-cell infiltration increased 7 days after treatment in the best-performing dose, this observation coincided with the tumor returning to exponential tumor growth, which suggests that the remaining tumor was able to recover and continue growing despite early immune cell infiltration. Increased prevalence of CD8^+^ T cells is generally associated with favorable outcomes in immunotherapy [30], but the B16F10 tumor model is known to be “immunologically cold” and contain weak natural antigens that may limit the effectiveness of antigen-specific responses [31,32]. Recent work from the Price lab has demonstrated that mechanical ablation directly led to cell-free tumor antigen accumulation and uptake by antigen presenting cells outside of the treated tumor in the tumor-draining lymph node (TDLN), which resulted in an antigen-directed anti-tumor response in the B16F10-ZsG tumor model [33]. The importance of antigen kinetics could partially explain the poor immune outcomes at the highest tested therapy dose (100 ppl). It is possible that excessive cavitation could degrade subcellular components, such as DAMPs or potential tumor antigens, diminish the “immunogenicity” of the tissue homogenate, and minimize/delay the anti-tumor immune response. High doses of histotripsy may negatively impact the kinetics of antigen presentation, either by decreasing the ability of dendritic cells to traffic from the tumor to the draining lymph node, or by decreasing cell-free antigen accumulation in the draining lymph node [34,35,36]. Previous studies have indicated that histotripsy induces an interruption of tumor hypoxia [37] and induction of necroptotic cell death [11], and it is possible that high doses could result in lower levels of these potentially immunogenic effects. This is only speculation, however, and experimental validation is necessary to understand the molecular content and reabsorption kinetics of the generated lysate, as well as assess changes to the tumor microenvironment if histotripsy aims to synergize with immunotherapy for improving patient health.

While this study has promising implications for the role of histotripsy dose in anti-tumor immune stimulation, there are several limitations. First, histotripsy monotherapy had a very modest effect on long-term B16F10 tumor growth. Previous studies using the B16F10 model have shown greater therapeutic benefit following histotripsy, with greater tumor growth inhibition and immune cell infiltration compared to no therapy [10,11]. While the cell line, inoculation methods, and targeting methods were identical between studies, an important difference to note is the difference in transducer power settings. In past studies, the focal pressure being delivered to the therapy transducer was variably adjusted during treatment to minimize cavitation near the skin surface and in muscle. In this study, pressure remained fixed at a high output to minimize confounding variables in histotripsy dose. This limited the targeting window for treatments due to a fixed bubble cloud size and decreased the proportion of the tumor that was delivered therapy. The use of a higher pressure output may have also led to unintended collateral damage to surrounding tissue, which could impact the local immune response [38]. Furthermore, unlike previous studies [10,11], we performed single treatments to all tumors and intentionally avoided additional treatments outside the planned treatment grid (as had previously been performed to ensure satisfactory treatment for irregularly shaped tumors). Although this was carried out to reduce confounding variables, it is quite possible that this resulted in a relative reduction in the volume of treated tumor as compared to previous studies.

Additionally, even though an increase in CD8^+^ density was measured in this study, it does not guarantee an effective cytotoxic response. Markers of cell proliferation with Ki67 [39], T-cell exhaustion with PD-1/PDL-1 [40], T-cell function with IFN-**γ** [41], or granzyme-B [42] could be used in combination with CD8^+^ density to provide context to their functional state. Future studies should utilize these cell markers and cytokines associated with immune outcomes within the tumor microenvironment and tumor-draining lymph node(s) to obtain a more complete picture of the dose-dependent immune response after histotripsy. Lastly, investigating lower histotripsy doses that induce less tissue fractionation in the targeted tissue could also provide insight into histotripsy immune stimulation after minimal tissue disruption.

## 5. Conclusions

This study demonstrates that histotripsy dose significantly impacts anti-tumor immune responses and therapeutic efficacy in a subcutaneous B16F10 melanoma model, with an intermediate dose (40 ppl) providing superior tumor growth inhibition and survival compared to the other tested doses. The non-monotonic relationship between treatment intensity and therapeutic benefit, where maximum benefit occurs at an `intermediate’ dose (40 ppl) instead of the highest dose (100 ppl), challenges the assumption that maximal tissue fractionation optimizes outcomes. This suggests instead that there is a `sweet spot’ for favorable immune stimulation. The observed early decrease in tumor-associated macrophages and subsequent increase in CD8^+^ T-cell infiltration at the optimal dose highlights the dynamic interplay between histotripsy treatment parameters and the tumor immune microenvironment at various timepoints. These findings highlight the importance of dose optimization in histotripsy therapy and suggest that balancing mechanical tumor destruction with preservation of immunogenic material may be critical for maximizing therapeutic efficacy. Future investigations into lysate composition, antigen bioavailability, and the mechanisms governing immune cell recruitment and function will be essential for rationally designing histotripsy regimens that synergize with immunotherapy approaches to improve clinical outcomes in cancer treatment.

## Figures and Tables

**Table 1:** Pathologist scoring metrics.

Scoring Metrics			
% Necrosis	Score	Residual Structure	Score
0%	0	None present	0
1–25%	1	Patchy small areas	1
26–50%	2	Larger patchy areas	2
51–75%	3	Diffuse areas	3
76–95%	4	Intact	4
>95%	5		

## Data Availability

The information is contained within this article in its entirety. For additional information, please feel free to inquire with either the original author or the corresponding author.
